# 

**Published:** 1887-02-12

**Authors:** 


					r V. 7. f PRICE ONE PENNY.
No. 20, Vol. I.
(February 12th, 1887.
0
Per Annum, 6s. 6d.,post free.
Monthly Parts, 6d.; post free, 7id.
Ditto per Ann., 7s. 6d., post free.
?pMBipl
|ii[UiJjPiE
liiUmiful
?Hi dsn
. ? - --??
JSTT^aSStfoSpl Cat  <-. -T rJ l.",r^c\ 1.
&n Institution, iTamitj?, an&^fc. $arocf)iaI Journal of
hospitals, asnlums, & all agencies for tfje dTare of tfje sMelt, Criticism & fietos.

				

